# Activation of the STING pathway enhances immunity and improves survival in a murine myeloid leukemia model

**DOI:** 10.1186/2051-1426-2-S3-P166

**Published:** 2014-11-06

**Authors:** Emily Curran, Xiufen Chen, Leticia Corrales, Justin Kline

**Affiliations:** 1 University of Chicago, Chicago, IL, USA

## 

Type I interferon (IFN) production by innate immune cells is critical to prime spontaneous T cell responses against solid tumors. Emerging pre-clinical data suggests that tumor-derived DNA induces potent IFN-β production by activating a cytosolic DNA sensing receptor called STING (Stimulator of Interferon Genes), ultimately resulting in tumor antigen-specific T cell priming and in some cases, tumor rejection. However, because of their disseminated growth pattern, hematological malignancies, such as acute myeloid leukemia (AML), may not release sufficient quantities of DNA during cell death to activate the STING pathway in innate immune cells, which contributes to the T cell tolerance observed in these hosts. Thus, we hypothesized that STING pathway activation would promote a host type I IFN response sufficient to facilitate leukemia-specific T cell priming and immune-mediated control of AML progression. Murine C1498 AML cells expressing the model SIY antigen (C1498.SIY) were inoculated intravenously into syngeneic C57BL/6 mice which then received a single dose of the murine STING agonist, DMXAA (5,6-dimethylxanthenone-4-acetic acid), or vehicle control 5 days later. DMXAA induced potent IFN-β production by C57BL/6 spleen cells (90-fold induction over vehicle control). STING pathway activation via DMXAA treatment of C1498.SIY-bearing animals resulted in a massive expansion (10-fold over vehicle control) of endogenous SIY antigen-specific T cells as analyzed by SIY/K^b ^pentamer staining and flow cytometry. Intracellular cytokine staining of SIY-peptide restimulated spleen cells from DMXAA-treated animals revealed that the expanded SIY-specific CD8^+ ^T cells produced high-levels of IFN-γ. This enhanced immune response also translated to a benefit in survival. After C1498.SIY AML cell-challenge, 80% of DMXAA-treated mice survived long-term, while controls succumbed to AML within approximately 3-4 weeks, as expected. DMXAA-treated mice surviving a primary C1498.SIY cell challenge also rejected a secondary challenge with parental C1498 cells (SIY-negative), suggesting DMXAA-induced STING activation led to potent immunological memory against native C1498-expressed antigens. DMXAA treatment of mice following a systemic challenge with parental C1498 cells also led to significantly enhanced survival compared to control-treated animals. This effect was T and/or B cell-dependent, as DMXAA treatment of leukemia-bearing RAG^-/- ^mice had no effect on survival. Collectively, these data suggest that DMXAA-mediated activation of the STING pathway enhances adaptive immunity to AML, culminating in prolonged survival and even disease cure. Human STING agonists are currently in development and, if STING pathway activation demonstrates efficacy in additional pre-clinical leukemia models, it may be of interest to target this pathway in AML patients.

